# The first complete chloroplast genome of *Hovenia dulcis* Thunb. (Rhamnaceae)

**DOI:** 10.1080/23802359.2021.1887772

**Published:** 2021-03-17

**Authors:** Dan Liu, Bo-qiang Tong, Wen-Qing Li, Lei Wang, Yang Xian, Biao Han, Xin Dong, Yi-Zeng Lu, Wei Li, Xiao-Man Xie

**Affiliations:** a College of Biological Sciences and Biotechnology, Beijing Forestry University, Beijing, People’s Republic of China; b Shandong Forest Germplasm Resources Center, Ji’nan, Shandong Province, People’s Republic of China; c Key Lab of Plant Stress Research, College of Life Sciences, Shandong Normal University, Ji’nan, Shandong, China

**Keywords:** *Hovenia dulcis*, chloroplast genome, phylogenetic relationship

## Abstract

The complete chloroplast genome of *Hovenia dulcis* was sequenced with Illumina HiSeq 2000 platform. It was a typical quadruple structure as other plants of *Hovenia* with 162,962 bp in length, including a large single-copy (LSC: 90,900 bp) region and a small single-copy (SSC: 18,920 bp) which were separated by a pair of inverted repeats (IRa, b: 26,571 bp) region. The overall GC content is 36.6%. A total of 130 genes was annotated which contained 85 protein-coding genes including the Trans splicing gene of *rps*12, 37 tRNA genes, and 8 rRNA genes. ML phylogenetic analysis compared with 6 expressed chloroplast genomes of Rhamnaceae revealed that *H. dulcis* was closely related to the species of *Zizyphus*, and which were clustered into a group with *Z. jujuba, Z. mauritiana* and *Z. spina-christi. Hovenia dulcis* was relatively distant to other species of *Berchemiella* and *Rhamnus*, which were clustered into another group.

*Hovenia dulcis* Thunb. is an important medicinal, ornamental, and timber tree species in Rhamnaceae, which are mainly distributed in Hebei, Shandong, Shanxi, Henan, Shaanxi, Gansu, northern Sichuan, Western Hubei, Anhui, Jiangsu and Jiangxi of China, as well as Japan and North Korea (Chen 1982). At present, the research of *H. dulcis* mainly focuses on breeding (Zhang et al 2009), the function of medicinal components (Tang and Zhu 2012) and UPLC fingerprint (Li and Liu 2015) and so on. However, there is no report on the genomics of *H. dulcis*. Genomics research will contribute to the development and utilization of *H. dulcis*.

The fresh leaves of *H. dulcis* were collected from the living individual permanently conserved in the *Hovenia* gene bank (36.633°N, 117.176°E) and its provenance was Laoshan Mountain in Shandong Province. The specimens were preserved in National Plant Specimen Resource Center (http://www.cvh.ac.cn/, barcode SDF1007434). Total genomic DNA (saved in DNA library of Shandong Forest Germplasm Resources Center with the code of bzj2020cp01) was extracted by the Plant DNA extraction Kit (TIANGEN, Beijing, China) according to the requirements of the reagent company.

Paired-end reads were constructed according to the Illumina library preparation protocol and sequenced on an Illumina HiSeq 2000 platform. The whole chloroplast genome of *H. dulcis* was assembled by MITObim v1.8 (Hahn et al. 2013) and was annotated in DOGMA (http://dogma.ccbb.utexas.edu/). The whole chloroplast genome of *H. dulcis* and other 6 published plastomes of Rhamnaceae were conducted by using MAFFT v7.429 (Katoh and Standley 2013) and with *Vitis amurensis* as outgroup. Maximum-likelihood (ML) phylogenetic tree with 1000 bootstrap replicates was inferred using IQ-TREE v1.6.12 (Lam-Tung et al. 2015) and TVM + F+R2 model.

The chloroplast genome of *H. dulcis* (GenBank accession number MT916772) was also a typical quadruple structure with 162,962 bp in length that contains a large single copy (LSC: 90,900 bp) region and a small single copy (SSC: 18,920 bp), which were separated by a pair of inverted repeats (IRa, b: 26,571 bp) region. The overall GC content was 36.6%. A total of 130 genes were annotated, including 85 protein-coding genes, 37 tRNA genes, and 8 rRNA genes. Among these, 15 genes had a single intron respectively, *clp*P and *ycf*3had two introns respectively. While *rps*12 had Trans splicing function. ML phylogenetic analysis compared with 6 expressed chloroplast genomes of Rhamnaceae revealed that *H. dulcis* was closely related to the species of *Zizyphus*, and which were clustered into a group with *Z. jujuba, Z. mauritiana* and *Z. spina-christi. H. dulcis* had relatively distant to other species of *Berchemiella* and *Rhamnus*, which were clustered into another group (Figure 1).

**Figure 1.**
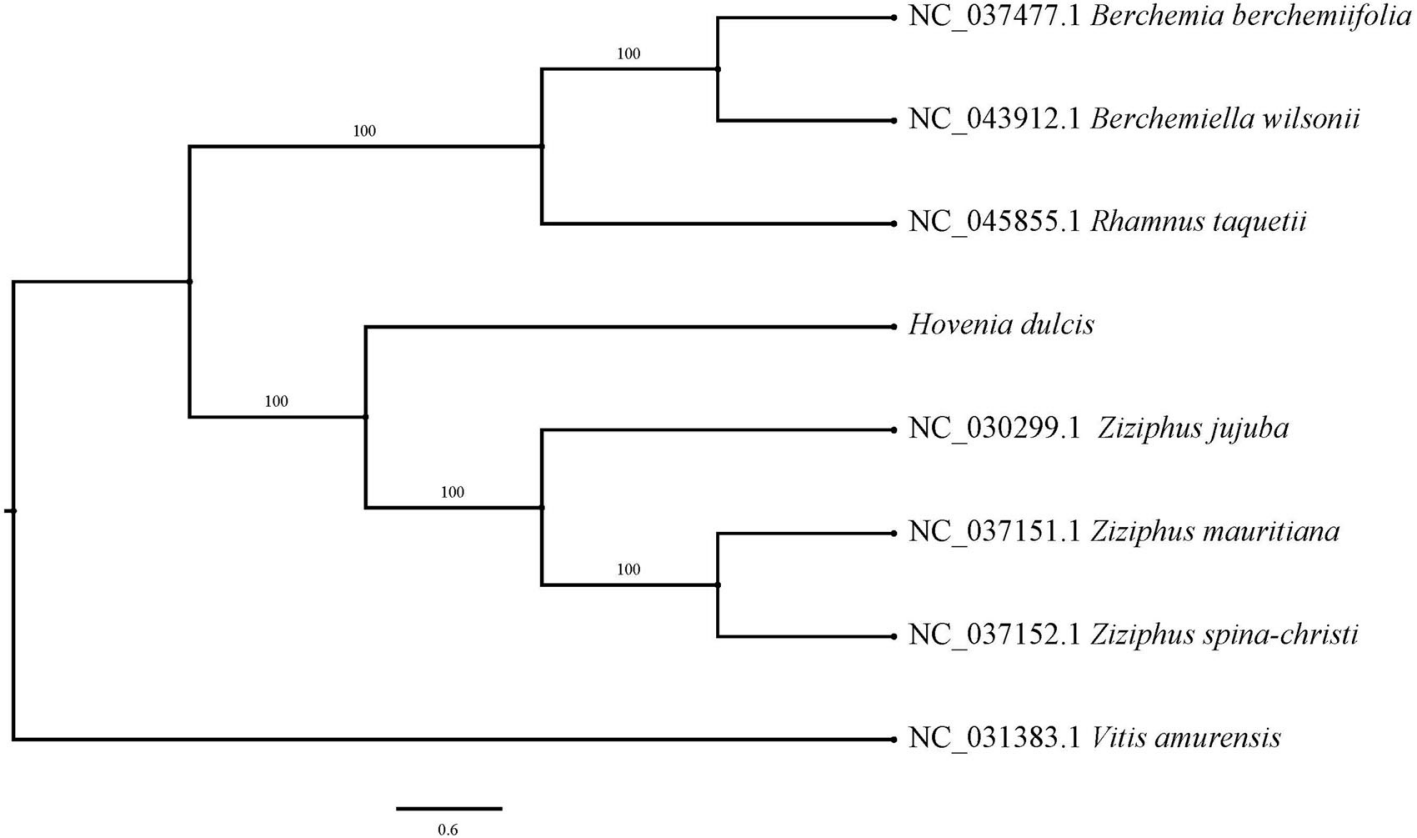
A maximum-likelihood (ML) tree of *Hovenia dulcis* and other 6 related species of Rhamnaceae based on the complete chloroplast genome sequences with *Vitis amurensis* as outgroup. The accession numbers are showed in the figure, and the numbers behind each node are bootstrap support values.

## Data Availability

The genome sequence data that support the findings of this study are openly available in GenBank of NCBI at (https://www.ncbi.nlm.nih.gov/) under the accession no. MT 916772. The associated BioProject, SRA, and Bio-Sample numbers are PRJNA694827, SRR13528763, and SAMN17574948 respectively.
